# Editorial: Perineuronal Nets as Therapeutic Targets for the Treatment of Neuropsychiatric Disorders

**DOI:** 10.3389/fnsyn.2022.889800

**Published:** 2022-06-10

**Authors:** Caroline A. Browne, Katherine Conant, Amy W. Lasek, Juan Nacher

**Affiliations:** ^1^Department of Pharmacology & Molecular Therapeutics, Uniformed Services University, Bethesda, MD, United States; ^2^Department of Neuroscience, Georgetown University Medical Center, Washington, DC, United States; ^3^Center for Alcohol Research in Epigenetics, Department of Psychiatry, University of Illinois at Chicago, Chicago, IL, United States; ^4^Neurobiology Unit, Program in Neurosciences and Institute of Biotechnology and Biomedicine (BIOTECMED), Universitat de València, Burjassot, Spain; ^5^Spanish National Network for Research in Mental Health, Centro de Investigación Biomédica en Red de Salud Mental (CIBERSAM), Madrid, Spain; ^6^Fundación Investigación Hospital Clínico de Valencia, INCLIVA, Valencia, Spain

**Keywords:** perineuronal nets (PNNs), PV interneurons, memory, microglia, multiple sclerosis, schizophrenia, therapeutics

Dysregulated synaptic plasticity and excitatory inhibitory (E/I) imbalance are molecular hallmarks of multiple neuropsychiatric disorders. Perineuronal nets (PNNs) are condensed portions of the extracellular matrix (ECM) that stabilize homeostatic synaptic plasticity, facilitate neuronal-glia communication, and provide a physical barrier protecting neurons from toxic molecules within the ECM (Carulli et al.; Wingert and Sorg). Pathological alterations in PNNs have been reported across several neuropsychiatric conditions including, but not limited to, post-traumatic stress disorder (PTSD), multiple sclerosis (MS), epilepsy, substance use disorders (SUDs), and schizophrenia. Thus, targeting the restoration of normal PNN function has emerged as a potential avenue for the development of therapeutics for the remediation of several diseases/disorders.

PNN complexity in function is also reflected in composition. The various components of PNNs can be modulated to dynamically alter plasticity under normal conditions and in disease. The lattice-like structures of PNNs are formed by the binding of chondroitin sulfate proteoglycans (CSPCs) to a hyaluronan backbone on a neuronal cell. Hyaluronan-CSPG binding is stabilized by link proteins, such as hyaluronan and proteoglycan link protein 1 (HAPLN1) and HAPLN4, and CSPGs have an overall negative charge. PNNs typically contain the lectican aggrecan, with varying levels of neurocan, versican and brevican (Fawcett et al., 2019), which are in turn linked together by the ECM glycoprotein, tenascin-R. The final critical component of the structure are the chondroitin sulfate glycosaminoglycan (CS-GAG) chains, at which a large array of molecules may bind, altering the local environment, modifying synaptogenesis and plasticity (Fawcett et al., 2019). Accordingly, there are numerous receptors, integrins and other adhesion/guidance molecules that interact with PNNs on a regular basis. Moreover, PNN integrity is regulated by a number of enzymes secreted by other cell types throughout the CNS. As patterns of aberrant PNN function continue to be linked to pathophysiology, it is enticing to propose therapeutic strategies that could potentially halt disease progression or restore function. The field is in its nascent stages, with several limitations that need to be surmounted, including adequate visualization, identification, and ECM component specific knockdown strategies. To this end, this editorial summaries some of the recent preclinical literature that highlight the potential of targeting PNN function to yield therapeutic outcomes.

## PNN Regulation of GABAergic Interneurons

PNNs predominantly enwrap the cell bodies and proximal dendrites of fast spiking parvalbumin (PV) positive GABAergic interneurons within the central nervous system (Wingert and Sorg). The fundamental role of PV-containing interneurons is to inhibit firing of local pyramidal neurons (Wingert and Sorg). Depending on the firing patterns of the inhibitory circuit, PV positive interneurons have the capacity to shape network responses to external stimuli. Wingert and Sorg critically review the impact of PNN removal from these circuits to determine the electrophysiological impact across multiple brain regions implicated in psychiatric disorders. Overall, it has been established that PV interneurons are highly excitable and most active during awake sensory processing. A single action potential efficiently recruits PV cell-mediated feedback inhibition on firing pyramidal neurons. That is, a single evoked or spontaneous action potential in a pyramidal cell will induce excitatory post-synaptic potentials (EPSPs) that generate an action potential in a connected PV positive interneuron. These properties allow PV interneurons to synchronize principal neuron output, but the exact motif of inhibition produced by PVs within local brain regions and circuits is still largely undefined. Summarizing the most consistent findings from the field, the evidence indicates higher action potential thresholds and a decrease in firing rate of fast spiking PV positive GABAergic interneurons after PNN removal (Wingert and Sorg). This can result in significant alterations at a circuit level, for example reduced hippocampal long-term potentiation (LTP) within the Schaffer Collateral pathway (Wingert and Sorg). At a functional level, this deficit manifests as impaired learning and memory performance, an important facet in multiple disorders and diseases.

## PNNs and Memory

In the context of disease, it is also important to note that PNN levels may be increased and attenuation of PNNs in that context can facilitate learning and cognitive flexibility (Duncan et al., 2019), highlighting the complexity of PNNs in regulating learning and memory. In related studies that examine PNN alterations that occur physiologically and in a relatively short time scale, aberrant sleep may dramatically alter memory consolidation in part through altered PNN structure (reviewed by Gisabella et al.). Indeed, it has been previously shown in mice that the PNN composition exhibits circadian rhythmicity. This includes elevated densities of *Wisteria floribunda* agglutinin (WFA)-labeled PNNs during arousal and lower levels of WFA during sleep in the hippocampus (Pantazopoulos et al., 2020) and prelimbic cortex (Harkness et al., 2019). Moreover, the amplitude of LTP and synaptic strength follows the diurnal pattern of PNN densities and the expression of chondroitin sulfate synthase 1 in particular, which exhibits peaks of expression at Zeitgeber time (ZT) 20 and troughs at ZT8, with ZT 0 starting at the onset of the light phase (Gisabella et al.). This pattern of decreased PNN composition during sleep coincides with decreases in synaptic downscaling and LTP, which may facilitate memory consolidation, processes which are blunted following sleep deprivation. Of interest are the coincident and entrained alterations in the expression of the transcription factor orthodenticle homeobox 2 (OTX2), glutamatergic NMDA receptors, matrix metalloproteinase 3 (MMP3) and 9 (MMP9) and cathepsin-S that occur with sleep deprivation, reviewed in Gisabella et al. With regards to PTSD, the importance of PNNs in the acquisition/retention of trauma associated memories have led many researchers to evaluate specific PNN components as therapeutic targets.

Building on their previous work, Lesnikova et al. evaluate the impact of receptor protein tyrosine kinase (PTP) activation by CSPGs binding in constraining tyrosine kinase 2 (TrkB) dependent neuronal plasticity. Previous studies have reported that deletion of PTPs increased phosphorylation of TrkB, thereby promoting plasticity within the visual cortex (Lesnikova et al., 2021) and that Ch-ABC-induced plasticity requires intact TrkB expression on parvalbumin (PV) interneurons within that circuit (Lesnikova et al., 2021). Extending this research (Lesnikova et al.) characterized the molecular profile of enhanced plasticity in haploinsufficient mice. PTPσ+/- mice, generated on a BALB/c background, exhibited a 50% increase in phosphorylated TrkB and an 25% increase in phosphorylated phospholipase C gamma 1 (PLCγ1) expression in the prefrontal cortex, hippocampus, and amygdala relative to wildtype controls, with no significant regional variation in expression (Lesnikova et al.). Overall, these molecular alterations should facilitate neuronal plasticity. At a behavioral level these haploinsufficient mice did not show altered behavioral performances when screened for exploratory activity on the elevated plus maze, marble burying behavior, locomotor activity or time spent immobile in the forced swim test (Lesnikova et al.). In contrast, long term consolidation of memory was robustly diminished for novel object recognition (discrimination index for the novel/familiar object) and contextual fear memory (% time spend freezing in the context) when assessed for recall at 5 and 10 days following the initial behavioral exposure, respectively. This contrasted with the increase in novel object discrimination of PTPσ+/– mice when tested 4 h following training (Lesnikova et al.). Although no direct alterations in the number of synapses were reported in these PTPσ+/– mice, there is evidence in the literature to support the supposition that PTPσ deficiency results in impaired LTP and EPSCs (Horn et al., 2012; Kim et al., 2020). Similar findings of reduced LTP and disrupted EPSCs were also reported when TrkB was overexpressed in mice (Koponen et al., 2004). These data highlight the potential of therapeutically targeting PTPσ/PNN interactions. Such therapeutics could restore the ability of PNNs to constrain TrkB dependent plasticity. Mimetics for PTP sigma have been developed and have shown efficacy in preclinical models of spinal cord injury (SCI) (Lang et al., 2015). Based on these data further research of PNN/receptor interactions (this includes but is not limited to class II A PTPs, leukocyte common antigen-related phosphatase (LAR), Nogo NgR1 and NgR2) in the context of memory are warranted and the development of mimetics to target these interactions is viable.

## PNNs and Microglia

Microglia are also known to be critical factors in the acquisition, consolidation and recall of memory (Morris et al., 2013; Cornell et al., 2022). Recent studies implicate microglia in degrading ECM composition (Gisabella et al.). In particular, microglia produce proteases, including MMP9 and cathepsin-S, which cleave CSPGs (Taishi et al., 2001; Pantazopoulos et al., 2020). Synaptic balance of primary hippocampal neurons was shown to be disrupted by direct microglia induced PNN depletion in a series of well-designed *ex vivo* studies (Wegrzyn et al.). Primary hippocampal astrocyte-neuron co-cultures and mixed glial cultures derived from the cortices of SV129 mice were utilized in medium exchange experiments to evaluate the impact of microglial activation by the toll like receptor 3 (TLR3) ligand Poly I:C on synapse formation and network activity (Wegrzyn et al.). Single occurring action potentials (spikes) were robustly elevated in all conditions when evaluated following 3 h of incubation. Increases in spike number persisted (up to 48 h) only in neurons treated with microglia conditioned medium relative to their non-microglial conditioned Poly I:C and hippocampal medium controls. The number and duration of burst firing of neurons was also significantly enhanced in microglia conditioned medium. The increased spontaneous network activity of neurons treated with microglia conditioned medium was not dependent on baseline spike frequency or impacted by apoptosis, and no specific subpopulation of neurons was identified as mediating this change in network activity. Wegrzyn et al. proceeded to demonstrate that PNN ensheathed neurons (aggrecan positive cells) treated with microglia conditioned medium exhibited robust disruption of PNN covering relative to the even covering of the soma and proximal dendrites of neurons by PNNs in the control medium groups (Wegrzyn et al.). These cells exhibited a robust decrease in formation of inhibitory but not excitatory synapses (vGAT and vGlut positive puncta co-localized with post-synaptic gephyrin and PSD-95 positive puncta, respectively), and a resultant shift toward higher excitatory input overall (Wegrzyn et al.).

These findings highlight the importance of microglia secreted factors in the regulation of PNN ensheathed neuron function in the context of inflammation. As microglia are known to have a pathological role across a spectrum of diseases and disorders, there is a clear rationale to pursue therapeutics that can negate the impact of secreted factors (such as proteases including cathepsin S) for a range of psychiatric and neurodegenerative disorders. This may involve evaluation of small molecule inhibitors such as morpholinurea-leucine-homophenylalanine-vinylsulfone-phenyl (LHVS) which is currently in clinical trials for the treatment of psoriasis.

## Therapeutic Potential for Multiple Sclerosis

There is growing evidence to support alterations in ECM components in the context of multiple sclerosis (MS) (for a comprehensive review of this literature see (Ghorbani and Yong, 2021). As PV+ axons are myelinated and activity dependent oligodendrocyte enwrapping of these interneurons is shaped by ECM, numerous studies are now exploring the regulation of oligodendrocyte/PNN interactions. (Lourenço et al., 2016; Gorter and Baron, 2020). Indeed, oligodendrocyte progenitor cells are enriched for PNN components including aggrecan, neurocan, versican, phosphacan, brevican and tenascin-R in a pathological state where PNNs were elevated (Tanti et al., 2022). It is therefore rationale to speculate that where oligodendrocytes are lost, PNNs may also be impacted. To our knowledge only two clinical studies have directly assessed the role of PNNs in the context of MS. Postmortem evaluation of cortical plaques suggests that MMP-9 dependent degradation of PNNs is associated with MS. In that report, sections of frontal and temporal cortex from nine patients with MS and seven healthy controls were screened for WFA+ and PV+ labeling, MMP-9, and myeloperoxidase (MPO) activity (Gray et al., 2008). Total and active MMP-9 levels were elevated in non-myelinated samples, relative to matched myelinated MS and control samples. Double labeling for MMP-9 and microtubule-associated protein 2, localized MMP-9 positive cells within and outside plaques and significantly lower WFA labeling around those cells relative to matched non-demyelinated areas of cortex (Gray et al., 2008). In demyelinated cortex there was an obvious loss of PNNs surrounding PV+ neurons relative to non-demyelinated MS and control cortex (Gray et al., 2008). Moreover, perikaryal accumulation of phosphorylated neurofilament protein was evident in the PNN lacking PV+ neurons in demyelinated cortex. In another study, alterations in basement membranes were evaluated in associated with MS lesions (Van Horssen et al., 2006). Increased deposition of ECM components including heparan sulfate proteoglycans (HSPGs), laminin, collagen type IV and fibronectin were evident within the brain parenchyma of active MS plaques. Moreover, elevated levels of transforming growth factor-β1 (TGF-β1), a major regulator of ECM protein production, was noted around the plaques and isolated to microglia (Van Horssen et al., 2006). In line with these clinical data, a preclinical study of myelin oligodendrocyte glycoprotein (MOG_35−55_)-induced experimental autoimmune encephalomyelitis (EAE) reported a pronounced loss of both perisomatic PV+ and PNNs in layer 2/3 of the primary somatosensory cortex and a compensatory increase in VGlut1 positive cells (Potter et al., 2016). Whether the loss of PNNs in demyelination is causal or correlation has yet to be determined but these data support further exploration of ECM and oligodendrocyte/PNN interactions in the context of MS.

PNNs may also act as a scaffold for inhibitors of synapse formation such as semaphorin A (Carulli et al.). The eight semaphorin classes, the importance of neuropilin (Npn) and plexin (Plxn) proteins in binding semaphorins and the known functions of Sema3A, 3F, 3G, 4C, 5A, and 7A in the adult brain are presented in a comprehensive review by Carulli et al. In terms of therapeutic development, inhibition of Sema3A (which is found in adult cortical PNNs) may yield the most compelling results in the treatment of MS. Consistent upregulation in the aforementioned semaphorins, Npn and PlxnA1, in human brains of individuals diagnosed with MS are suggestive of a possible pathophysiology maker of MS. These changes are significant given that semaphorins are known to inhibit axonal regeneration, oligodendrocyte progenitor cell differentiation and oligodendrocyte migration. Utilizing murine models of MS, Biname and colleagues demonstrated that inhibition of Sema3A through local microinfusion of a peptide that antagonized Npn-1-PlxnA1 dimerization, effectively facilitate remyelination and rescue of MS associated motor deficits in murine models of the disease (Biname et al., 2019). These data are reviewed and evaluated in Carulli et al., and align with upregulation of other semaphorins in Amyotrophic Lateral Sclerosis and Alzheimer's Disease. Although these data support the direct targeting of Sema3A, a great deal of work is required to determine whether other PNN associated molecules have a role in modulating myelination in the context of disease.

## Targeting PNNs in Schizophrenia

Carulli et al. also evaluated the evidence supporting a role for semaphorins in the pathophysiology of schizophrenia. CSPGs in the amygdala and entorhinal cortex are known to be decreased in subjects with schizophrenia (Pantazopoulos et al., 2010, 2015). Furthermore, genome-wide association studies identified single nucleotide polymorphisms (SNPs) in the PlxnA2 gene which diminished PlxnA2 association with CSPGs in schizophrenia (Mah et al., 2006; Takeshita et al., 2008), but these findings have not been replicated in other cohorts with the same SNPs (Fujii et al., 2007; Budel et al., 2008). Although elevated levels of Sema3a, 4Da and PlxbB1 have been shown in the brains of human subjects with schizophrenia, there are also marked decreases in PlxnA1 and Sema3D (Carulli et al.), suggesting an inconsistency across data sets. Overall it is suggested that carrying a mutation in PlxnA2 may confer an added risk of developing this debilitating psychiatric disorder (Carulli et al.). These data support the need for more comprehensive evaluation of the PNNs and semaphorins in particular in the precipitation and treatment of schizophrenia. Indeed, it would be extremely worthwhile to determine whether alterations in PlxnA2 are associated with either the positive, negative, or cognitive symptoms of schizophrenia.

Emerging preclinical evidence supports a role of PNNs in mediating the aberrant synaptic plasticity and behavioral profiles associated with murine models of schizophrenia. Klimczak et al. extend their previous findings and detail alterations in PV expressing interneurons within the hippocampus and retrosplenial cortex of adult male FVB mice exposed to a “double hit” model (DHM) of schizophrenia. In this case, DHM was comprised of early life NMDA antagonism [MK-801 administered at post-natal day 7 (P7)] paired with social isolation (starting on P21), to mimic the pathophysiological disruptions observed in humans. Previously, the Nacher group has characterized behavioral deficits and altered plasticity in the amygdala of mice exposed to DHM, specifically identifying decreased PV expressing neurons and PNN expression in amygdala of these mice (Gilabert-Juan et al., 2013; Castillo-Gomez et al., 2017; Garcia-Mompo et al., 2020). Klimczak et al. report reductions in the number of PV positive somas surrounded by PNNs and a reduction in the area of the soma covered by PNNs in the CA1 region of the hippocampus in DHM exposed animals in adulthood (P90) relative to isolation and MK801 treatment alone. Of interest was a marked reduction in the number of PNN positive/PV negative somata in this region following isolation alone (Klimczak et al.). Within the retrosplenial cortex, a marked reduction in overall number of PV positive cells was evident in all treatment groups relative to vehicle control socially housed mice. PV positive neurons surrounded by PNN exhibited a marked reduction in the isolation alone group (Klimczak et al.). These regions are important in spatial working memory, a cognitive domain consistently impaired in patients with schizophrenia (Park and Holzman, 1992; Piskulic et al., 2007). The loss of PNNs and PV positive interneurons within these regions may contribute to overall excitatory/inhibitory imbalance, which is widely accepted as a possible etiopathology of schizophrenia. The DHM and similar double hit models provide a useful platform from which to interrogate potential PNN targeted therapeutics to alleviate the cognitive symptoms associated with schizophrenia.

## Future Directions for PNN Targeted Therapeutics

There are several limitations to our current understanding of PNN physiology and function. Optimization of PNN visualization remains a concern, for example not all aggrecan-containing PNNs are labeled with WFA. A recent review by Hartig and colleagues has outlined the conditions under which WFA staining is compromised, and detail strategies for optimized visualization (Hartig et al., 2022). In addition, the varied expression/overlap of tenascins and lecticans across brain regions could impact circuit specific function and thus warrant continued study (Ueno et al., 2017; Yamada and Jinno, 2017; Jakovljevic et al., 2021). Moreover, the importance of critical periods for PNN formation (Horii-Hayashi et al., 2015; Rogers et al., 2018; Ueno et al., 2018; Sigal et al., 2019) and the circadian rhythmicity of PNN density and composition (Gisabella et al.) are important factors that should be considered when designing future experiments.

To degrade PNN associated GAGs and CSPGs, current strategies rely heavily on Ch-ABC, a polysaccharide lysase derived from the bacterium Proteus Vulgaris. The level of CSPG degradation is limited by Ch-ABC's profile of thermal instability, short half-life and repeated dosing requirements to maintain therapeutically relevant knockdown of PNNs (Muir et al., 2019). Numerous groups have addressed this issue, stabilizing the protein either by site directed mutagenesis or by covalent molecules, such as glycerol, sorbitol or polyethylene glycol (PEGylation), which improved the half-life, aggregation and duration of PNN removal from the site of action (Hettiaratchi et al., 2019; Takashima et al., 2021; Wang et al., 2021). Local delivery of stabilized Ch-ABC has shown the greatest success in preclinical models of spinal cord injury (SCI) (Muir et al., 2019). Viral delivery of Ch-ABC has also been readily employed for rescue of SCI associated pathology *ex vivo* and *in vivo* (Burnside et al., 2018; Warren et al., 2020). Alternate strategies have combined stabilized Ch-ABC with scaffolds in efforts to improve long term targeted knockdown. This includes immobilized Ch-ABC I on dextran-coated Fe3O4 nanoparticles, this combination yielded an excellent kinetic profile (Askaripour et al., 2020). Similar stability and activity was achieved for at least 1 week with Ch-ABC coated gold nanorods (Naderi et al., 2018). Ultimately these biocompatible complexes will be used in conjunction with magnetic resonance imaging (MRI), photothermal or near infra-red (NIR) applications for guided knockdown of PNNs. Utilizing tamoxifen inducible selective Ch-ABC expression in hippocampal CA2 neurons in a conditional CA2 Cre-expressing mouse line, sustained knockdown of PNNs was achieved (Carstens et al., 2021). However, the knockdown was also evident in CA1 and CA3, with more widespread knockdown occurring with repeated tamoxifen injections. The field is now moving toward the use of targeted knockdown of PNN components by pharmacological or genetic means and away from the use of non-selective knockdown of PNNs by Ch-ABC administration. Such information will determine the level of contribution these PNN subcomponents confer to PNN function and synaptic plasticity in specific disease models and lead to the development of small molecule inhibitors or novel drug classes that can be developed clinically.

Overall, given the limitations in our current understating of ECM and PNN function, development of druggable targets for the remediation of neuropsychiatric disorders may be years off. The data presenting herein support the continued optimization of strategies for identification, visualization and therapeutically relevant knockdown of PNN components in a wide range of diseases/disorders.

## Author Contributions

CB, KC, AL, and JN wrote the editorial. All authors contributed to the article and approved the submitted version.

## Funding

JN is supported by RTI2018-098269-B-I00 financed by the Spanish Ministry of Science and Innovation/AEI/10.13039/501100011033/ (“FEDER Una manera de hacer Europa”) and the Generalitat Valenciana (PROMETEU/2020/024). KC is supported by R01NS10881. CB is supported by Transforming Technology for the Warfighter HU0001-20-2-0014 and Congressionally Directed Medical Research Programs PRMRP award W81XWH-21-2-0011.

## Conflict of Interest

The authors declare that the research was conducted in the absence of any commercial or financial relationships that could be construed as a potential conflict of interest.

## Publisher's Note

All claims expressed in this article are solely those of the authors and do not necessarily represent those of their affiliated organizations, or those of the publisher, the editors and the reviewers. Any product that may be evaluated in this article, or claim that may be made by its manufacturer, is not guaranteed or endorsed by the publisher.
